# Exploring Sex Differences in Pain Manifestation of Coronary Artery Disease through Mendelian Randomization

**DOI:** 10.3390/jcdd11090264

**Published:** 2024-08-27

**Authors:** Ruben Methorst, Monique R. M. Jongbloed, Raymond Noordam, Marco C. DeRuiter

**Affiliations:** 1 Department of Anatomy and Embryology, Leiden University Medical Centre, 2300 RC Leiden, The Netherlands; r.methorst@lumc.nl (R.M.);; 2 Department of Cardiology, Leiden University Medical Centre, 2300 RC Leiden, The Netherlands; 3 Department of Internal Medicine, Section of Gerontology and Geriatrics, Leiden University Medical Centre, 2300 RC Leiden, The Netherlands

**Keywords:** Mendelian randomization, cardiovascular disease, sex differences, pain, genetics

## Abstract

Pain manifestation following coronary artery disease (CAD) disease differs between men and women. Here, we aimed to provide evidence favoring possible differences in pain manifestation between men and women following CAD using Mendelian randomization (MR). We used summary-level data from sex-stratified genome-wide association studies on CAD and self-reported and clinically diagnosed chest, neck and shoulder, back, and facial pain using data from the UK Biobank cohort (*N* > 450,000) followed by two-sample MR (sensitivity) analyses. We identified 32 and 19 independent genetic variants associated with CAD for men and women, respectively, as instrumental variables. Genetically influenced CAD was associated with a higher risk of self-reported chest pain in both men (OR: 1.27, CI: 1.2–1.33) and women (OR: 1.44, CI: 1.20–1.73), with similar results for clinically diagnosed chest pain (men OR: 1.22, CI: 1.17–1.26; women OR: 1.31, CI: 1.18–1.46). In addition, in women only, genetically influenced CAD was associated with a higher risk of back pain (OR: 1.35, CI: 1.03–1.66) and neck and shoulder pain (OR: 1.22, CI: 0.91–1.63) (*p*-values for interaction with men: 0.030 and 0.041, respectively). Sensitivity analysis did not indicate the results were biased by directional pleiotropy. We found evidence, based on genetic predisposition for CAD, for different pain manifestations of CAD in men and women. While CAD was associated with chest pain in both sexes, we only found evidence for a higher risk of back pain and neck and shoulder pain in women, supporting common notions that women may present more often with uncharacteristic anginal symptoms.

## 1. Introduction

Coronary artery disease (CAD) is still the most common cause of mortality and morbidity in the Western world, despite well-accepted strategies for primary prevention [1]. Although the occurrence of CAD is dominated by men, in recent decades, it has become increasingly clear that women are also at risk, especially after menopause [2,3]. At the same time, clinical recognition of CAD in women has been described as more challenging than in men [4]. Several clinical reports suggest that while men often present with classic symptoms, i.e., chest pain radiating to the left arm and jaw, women may more often present with a broader range of less characteristic symptoms, including back and shoulder pain [4,5,6,7].

Evidence for sex differences in pain manifestations of cardiovascular disease is mainly derived from relatively small cohort studies with contradictory findings. While some studies identify distinct differences in pain manifestation [6,8], others do not find significant differences between sexes [9,10,11,12]. In addition, studying differences in pain manifestation between the sexes in traditional cohort studies is challenging for large groups of men and women, as this requires detailed knowledge of the pain manifestation of cardiovascular disease (a subjective measure).

Mendelian randomization (MR) is a technique that takes advantage of genetic variants encountered in large patient cohorts to identify specific associations between symptoms (e.g., chest pain) and disease (e.g., CAD) [13]. An advantage of MR compared to traditional cohort studies is the use of genetic variants associated with the exposure of interest as instrumental variables, which do not change in an individual’s lifespan and are not affected by external factors. Successful MR studies provide evidence favoring a possible causal association in the absence of confounding and reverse causality [14]. Large biobanks, such as the UK Biobank (UKBB), which includes approximately 450,000 European-ancestry participants, provide genetic information about participants to support such genetic association studies on a large scale. Previous MR studies in this field have commonly focused on the risk factors for CAD [15,16]. In the current study, MR is used to investigate whether there is genetic evidence that pain is caused by CAD (i.e., pain manifestation as a result of CAD onset).

Here, we aimed to provide evidence favoring possible differences in pain manifestation between men and women following CAD using MR.

## 2. Materials and Methods

### 2.1. Study Design and Study Population

The UKBB is a prospective cohort of participants aged between 40 and 69 years old at baseline, which was included between 2006 and 2010. At baseline, self-reported questionnaires were filled out about the lifestyle and medical history of participants. Hospital inpatient data were gathered and included more than 450,000 participants. Data are encoded using the World Health Organization’s ICD codes (International Classification of Diseases and Related Health Problems). For the genotyping of the participants, blood samples were collected.

The UKBB received approval from the NHS North-West Multi-Center Research Ethics Committee. All participants provided electronically written informed consent [17]. The present study was accepted by the UKBB under project number: 56340.

### 2.2. Ascertainment of Exposure and Outcome Variables

Chest pain was included in both self-reported chest pain and clinically diagnosed chest pain. Self-reported chest pain was a reported answer to the standardized question, “Do you ever have any pain or discomfort in your chest?”. Clinically diagnosed chest pain was extracted from hospital inpatient data: Main ICD-10 R07.4 Chest pain: unspecified. Main ICD-10 Diagnoses were chosen over Secondary ICD-10 Diagnoses to prevent the inclusion of patients also experiencing the exposure trait (CAD) and minimize sample overlap.

Other pain types commonly associated with CAD manifestation (and present in the UKBB) were included using pain experience last month: “In the last month, have you experienced any of the following that interfered with your usual activities? Pain types previously reported to be associated with CAD were included in the analysis: facial, neck and shoulder, and back pain [4]. UKBB traits dichotomize the presence of pain, while pain can be defined as a continuous factor using pain scales. In this study, we assume the monotonicity assumption due to our incentive for distinguishing subgroups of pain localization for MR [18].

CAD was defined using disease incidence data integrated from hospital admissions data and national death register data according to the ICD coding (recruitment to 1 January 2021) [17]. CAD was defined using ICD-10 codes (first occurrence): angina pectoris (I20), myocardial infarction (I21 and I22), and acute and chronic ischemic heart disease (I24 and I25). Control participants were participants without a date of diagnosis for the stated inclusion criteria.

### 2.3. Sex-Stratified Genome-Wide Association Analysis

Genome-wide association studies (GWAS) were performed in men and women separately for the exposure trait (CAD) and all outcome (pain) traits on autosomal chromosomes only. A linear mixed model was used to perform phenotype and genotype association testing with the BOLT-LMM algorithm v2.3beta2 [19]. Self-reported non-European ancestry participants were excluded. GWAS were adjusted for age, the first 10 genetic principal components, and the genotyping array. Additionally, GWAS were corrected for a familial relationship using the genetic correlation matrix. SNPs with a minor allele frequency < 0.01 were excluded, as were SNPs with an imputation quality < 0.3.

Significant GWAS hits (*p*-value < 5 × 10^−8^) were clumped (R^2^ < 0.001) to identify significant independent lead SNPs. Gene mapping, according to the NHGRI-EBI GWAS Catalog, was acquired using the closest-features (<50 kb) function of BEDOPS v2.4.40 [20]. dbSNP v153 was used for genetic variant calling, and GENCODE release 40 [21] was used for gene location calling on GRCh37.

### 2.4. Mendelian Randomization Analyses

Two-sample MR was performed using the summary statistics of the sex-stratified GWAS associations using the R-package TwoSampleMR [22]. Genetic variants associated with the exposure traits (CAD) with a *p*-value < 5 × 10^−6^ were clumped with a maximum linkage disequilibrium (LD) R^2^ < 0.001 to ensure independence of instrumental variables (IVs). The threshold of 5 × 10^−6^ was chosen to allow more genetic variants to be used as genetic instruments in the MR while still having a sufficient F-statistic (>10) to prevent weak-instrument bias. F-statistic > 10 was considered a strong instrument for analysis (F-statistic = R^2^ (n − 1 − k)/(1 − R^2^) k) [23]. Palindromic alleles were handled using allele frequency information. Inverse variant weighted (IVW) results were considered the main outcomes.

Assumptions of MR are the following: (i) genetic variants are associated with the exposure (CAD), (ii) genetic variants only influence the outcome (pain) via the exposure, and (iii) genetic variants are independent of any confounding factors [13].

MR-Egger (intercept) and a weighted median estimator were used to assess horizontal pleiotropy. A non-zero MR Egger intercept represents the presence of pleiotropy, indicating that IVW results might be biased. Cochran’s Q-value was calculated using the mr_heterogeneity() function and default parameters to estimate potential heterogeneity.

Additionally, the R-package MR-PRESSO [24] was used to detect and exclude outlying genetic instruments that could potentially bias the summary estimate using default parameters and NbDistribution = 3000.

The directionality of a possible causal effect between exposure and outcome, as well as individual IVs showing effects in the other directionality, were assessed using the Steiger test [25]. Leave-one-out analysis enabled the assessment of specific variants driving the whole effect, indicating invalid IVs.

Due to the nature of the UKBB, we can expect potential sample overlap between our exposure and outcomes; however, bias introduced due to sampling overlap is generally indispensable compared to any other biases, especially in large samples [26].

MR estimates were converted to odds ratios (ORs), and standard errors were converted to 95% confidence intervals (CIs). An interaction *p*-value was calculated using the differences between the estimates (beta) and standard errors to determine whether the estimate was different between men and women [27]. MR power analysis was performed using the mRnd binary outcome online tool [28]. The error rate (α) was set at 0.05, and the expected true effect was adjusted to find the required effect size given our sample size.

## 3. Results

### 3.1. Sex-Stratified GWAS Reveals Genome-Wide Significant Variants for CAD

**Figure 1:**
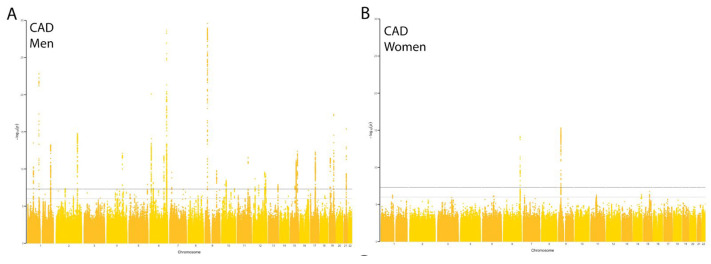
Manhattan plots of sex-stratified GWAS on CAD. Manhattan plots of summary statistics of GWAS results for CAD on men (**A**) and women (**B**). Each dot represents an individual genetic variant and its association with a given trait (−log(*p*-value)) on its position across the genome. The horizontal gray line indicates the suggestive threshold and MR threshold (*p*-value: 5 × 10^−6^). The horizontal black line represents genome-wide significance and the MR threshold at the *p*-value of 5 × 10^−8^.

A total of 22,323 men and 10,124 women were diagnosed with CAD (Supplemental Table S1). The total control population size is 411,007 (44% men). We performed a sex-stratified GWAS, revealing 33 and 3 independent genome-wide significant loci (*p*-value: 5 × 10^−8^, R^2^ < 0.001) for CAD in men and women, respectively (Figure 1A,B).

Next, we compared genome-wide significant loci for pain localizations between men and women (Supplemental Table S2). A handful of genetic variants showed a genome-wide significant association at a *p*-value of 5 × 10^−8^ in men and women. Self-reported chest pain showed a single locus passing genome-wide significance for men only. The locus is situated in a large intergenic region with a long non-coding RNA (ENSG00000284650) within 10 kb. The sole locus associated with clinical chest pain in men is the *LPA* locus. In women, this locus was within the suggestive significance threshold (*p*-value < 5 × 10^−6^). The other pains share loci with their non-stratified GWAS equivalents, such as the *CA10* locus in neck and shoulder pain in women. The *SOX5* locus in back pain in women is associated with multisite chronic pain in a sex-stratified setting, showing a women-specific direction of effect [29].

### 3.2. Mendelian Randomization Provides Evidence Favoring a Causal Effect between CAD and Chest Pain in Men and Women

A total of 104 independent IVs (R^2^ < 0.001) were included for men (GWAS *p*-value < 5 × 10^−6^, F-stat > 10) and 20 IVs (GWAS *p*-value < 5 × 10^−6^, F-stat > 10) for women (Supplemental Table S3). Given the fact that the number of CAD cases in women was two-fold smaller than in men and power analyses revealed relatively low power in GWAS analysis (Supplemental Table S4), we lowered the IV inclusion threshold to 5 × 10^−6^ for both analyses, provided that subsequent sensitivity analyses passed. The genetic variant associated with the *LPA* locus was removed in both men and women due to the likely introduction of horizontal pleiotropy (Supplemental Table S5). Finally, 103 IVs for men and 19 IVs for women were used for MR.

**Figure 2:**
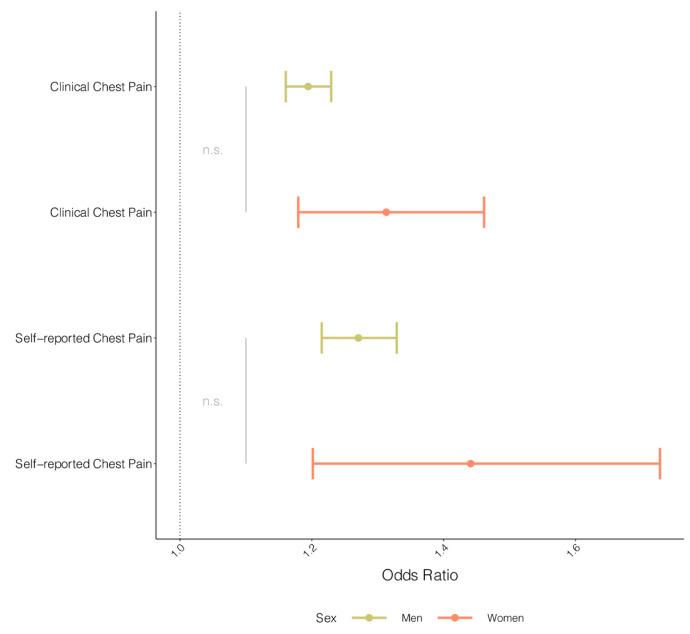
Sex-stratified MR of CAD on chest pain. The odds ratio of MR results for men (green) and women (red) on clinical chest pain (ICD10 R07.4) and self-reported chest pain from the UKBB cohort on CAD. The odds ratio was derived from IVW regression results. Error bars depict 95% confidence intervals of the odds ratio. Gray lines and text indicate the significance (n.s.: not significant) of the interaction test *p*-value.

We found evidence for an association between genetically influenced CAD and self-reported chest pain for both men (OR: 1.27, CI: 1.21–1.33) and women (OR: 1.44, CI: 1.20–1.73) (Figure 2), without evidence of possible effect differences between men and women (*p*-value for interaction = 0.189). Similar results were observed for the clinical diagnoses of chest pain (OR men: 1.22, CI: 1.17–1.26; OR women: 1.31, CI: 1.18–1.46) (Figure 2), with no evidence for differences detected between men and women (*p*-value for interaction: 0.19).

Sensitivity analyses revealed no evidence for the presence of potential horizontal pleiotropy and heterogeneity (Supplemental Table S6, Supplemental Figure S1).

### 3.3. Assessment of Sex-Specific Pain Localization of Coronary Artery Disease

**Figure 3:**
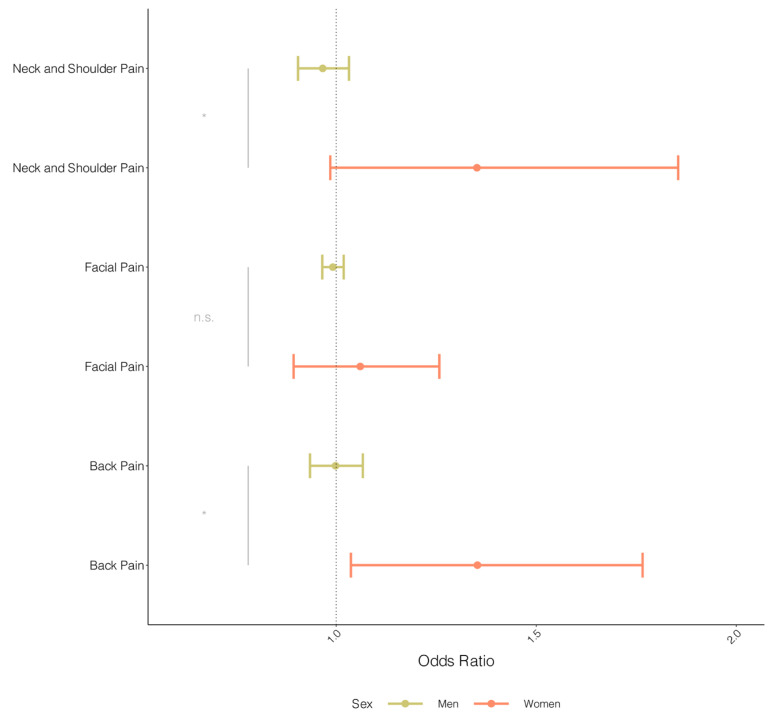
Sex-stratified MR of CAD on other pain types. The odds ratio of MR results for men (green) and women (red) on back pain, facial pain, and neck and shoulder pain from the UKBB cohort on CAD. The odds ratio was derived from IVW regression results. Error bars depict 95% confidence intervals of the odds ratio. Gray lines and text indicate the significance (n.s.: not significant, asterisk: *p*-value < 0.05) of the interaction test *p*-value.

The IVW MR analyses revealed no evidence for an association between genetically influenced CAD and the different pain manifestations in men: back pain (OR: 0.99, CI: 0.93–1.07), neck and shoulder pain (OR: 0.97, CI: 0.90–1.03), and facial pain (OR: 0.99, CI: 0.97–1.02) (Figure 3). IVW MR analyses in women revealed evidence of an association between genetically influenced CAD and back pain (OR: 1.35, CI: 1.03–1.66) but not with facial pain (OR: 1.03, CI: 0.88–1.21). In addition, we observed a trend toward genetically influenced CAD with neck and shoulder pain (OR: 1.22, CI: 0.91–1.63) in women but acknowledged the wide confidence intervals due to low(er) statistical power due to a low number of cases. When comparing the estimates between men and women, we obtained statistical evidence that the IVW estimates were different between men and women (*p*-values for interactions: 0.030 and 0.041, respectively, for back pain and neck and shoulder pain). MR sensitivity analyses revealed no evidence for potential pleiotropy or heterogeneity for all inferences in men and women (Supplemental Table S5, Supplemental Figure S1).

## 4. Discussion

In this study, we identified loci associated with CAD and sex-specific loci associated with chest pain, neck and shoulder pain, back pain, and facial pain. Our key findings provided evidence for a potential relationship between CAD and the manifestation of chest pain in both men and women (*p*-value < 0.001). Specifically for women, we showed genetic evidence for an increased manifestation of back pain (*p*-value: 0.026) upon CAD, with a similar trend for neck and shoulder pain (*p*-value: 0.062).

There was a lack of difference in the risk estimate of chest pain in women compared to men following CAD (*p*-value: 0.19). This is of particular interest as chest pain (historically regarded as a typical symptom of cardiovascular disease) has been proposed to be more dominant in men than in women [6,30]. However, contradictory studies exist; a plethora of clinical studies have also indicated that women are just as likely to experience chest pain as a manifestation of CAD as compared to men [9,10,11,12,31]. The latter is in line with the results from the MR analyses in the present study.

For pain locations other than the chest, we showed genetic evidence for an increased manifestation of back pain upon CAD in women compared to men at the population level, which aligns with the literature [6,8]. Neck and shoulder pain revealed a similar trend. A prospective cohort study of acute myocardial infarction patients (N = 903) showed that women reported pain in the neck and back more frequently than men, consistent with our findings [32]. Facial pain revealed no causal link with CAD in both sexes, despite jaw pain being a strong factor in CAD diagnosis in men and a more frequent occurrence of craniofacial pain in women [33]. To the best of our knowledge, this is the first MR-based study to infer disease manifestation using genetic variants and to study CAD pain manifestation at this large scale in a sex-specific manner.

The sensation of cardiac or thoracic pain is often the first sign that makes patients aware of a cardiac ischemic event. Next to thoracic pain, other pain locations are associated with the occurrence of ischemic damage. The heart responds to ischemia via nociceptive sensory neurons [34,35] that carry stimuli to the spinal cord. There, convergence with somatic sensory neurons occurs in the dorsal horn of the spinal cord (viscerosomatic convergence), and this is experienced by the patients as referred to in pain [36]. Referred cardiac pain is, therefore, dependent on the spatial distribution of the cardiac sensory neurons over the spinal segments [37]. Anatomical variation between individuals in projections and segmental localization of sensory neurons is of interest in light of inter-individual variations in cardiac pain perception. In this study, we showed evidence for potential underlying differences in anatomical variation between sexes regarding cardiac pain perception.

A lack of specification of the pain experience of individuals is observed, and the chest pain etiology encountered varies dramatically depending on the setting of patient presentation [38]. For instance, cardiac causes of chest pain are less commonly encountered by the general practitioner (20%) than by the ambulance dispatch crew (69%) and emergency department personnel (45%). However, these settings do not report differences in intensity, localization, or type of chest pain. A cohort study in the Netherlands reported that a mere 6% of patients seeking medical help for chest pain at the general practitioner’s office were ultimately diagnosed with acute myocardial infarction [39]. In acute medical care, about 25% of total cases are chest pain-related, of which the majority have confirmed or suspected acute myocardial infarction [40]. There is a patient-driven decision factor between requiring acute medical care and seeking non-acute care, most likely based on (chest) pain experience. A better understanding of CAD-related pain experiences is required to improve symptom recognition and awareness among the population.

In the current study, similar inferences for self-reported and clinically diagnosed chest pain were observed. We believe a key element between clinical and self-reported chest pain is the severity of the pain. Most likely, self-reporting participants answering “yes” to a given question display slight chest pain or sporadic chest pain, while clinically diagnosed chest pain characterizes participants who sought (acute) medical help for chest pain symptoms. This could be similar to the difference in chest pain etiology between a decision to visit a general practitioner and seeking acute medical care in a hospital. Interestingly, in our analyses, self-reported and clinical chest pain both showed a suggestive link with CAD. In-depth characterization of pain, such as the inclusion of pain scales, and more precise definitions of types of pain, such as differences between sharp and diffuse pain, are currently lacking in cohort studies. These data are required to increase the specificity of MR analyses linking disease with disease manifestation and, ultimately, to improve clinical diagnosis.

A particular genetic variant within the *LPA* locus, a known locus in cardiovascular disease, introduced pleiotropy in our analyses and was removed as a potential outlier, as it was identified in both GWAS on chest pain and CAD. This potentially suggests a shared common mechanism between chest pain and CAD via likely indirect effects [41].

Statistical power analysis revealed a potentially low sample size concerning neck and shoulder pain in women and facial pain for both sexes (Supplementary Table S4). Pain is a multi-causal trait that can be caused by many underlying aspects. Additionally, pain can occur incidentally, regularly, or chronically. Due to the nature of the data coding, it is hard to distinguish and exclude individuals experiencing chronic pain. Extrapolating the pain caused by CAD from this broad spectrum requires large sample sizes, which are currently only facilitated by the UKBB. Yet, the definition of facial pain in the UKBB may be too broad to embody the subset of CAD-related pain, i.e., radiating pain to the jaw. In our cohort, participants experiencing chronic pain could have been included in the analysis. A recent study explored the effect of multisite chronic pain on CAD. In that study, their reverse MR analysis revealed no significant relationship between CAD and multisite chronic pain [42]. Our results could nevertheless have been dampened by a subset of participants experiencing multisite chronic pain; however, our sensitivity analyses revealed no indication for this. Future studies require more elaborate pain ascertainment to increase the specificity of MR analysis. A prospective study in which a cohort of CAD patients is studied at baseline could potentially provide additional insights and be used for the purpose of validating the present results. Furthermore, it should be noted that the present study was conducted with participants of European ancestry only; extrapolation of this study’s results to other population groups should be performed with caution.

## 5. Conclusions

The present study provided evidence supporting a possible causal association between CAD and chest pain in men and women using MR, as well as a higher risk for women experiencing CAD to manifest with neck, shoulder, and back pain compared to men. Further study is required to investigate the intrinsic causes of these differences in cardiac pain perception between men and women.

## Data Availability

UKBB data are only available via the UKBB portal upon approval of a project plan and payment of an access fee. The present project was accepted by the UK Biobank under project number: 56340. Summary statistics of sex-stratified GWAS are available upon request. All data required for MR analysis are present in this manuscript. Scripts are available on GitHub: https://github.com/rubenmethorst/Sex-stratified-MR-CAD-Pain.

## Supplementary Materials

The following supporting information can be downloaded at: https://www.mdpi.com/article/10.3390/jcdd11090264/s1; Supplementary Materials include full MR and sensitivity analysis results; Figure S1: Leave-one-out plots for CAD inference testing stratified for sex, Table S1: Sample sizes of the different traits, Table S2: Genome-wide significant loci of different pain localizations stratified for sex, Table S3: Instrumental variables of CAD for men and women, Table S4: Results of power analysis, Table S5: Example of pleiotropy induced by a genetic variant in the LPA locus, Table S6: MR analyses and sensitivity analyses results of sex-stratified causal inference.
