# Supplementary material for: Exploring Sex Differences in Pain Manifestation of Coronary Artery Disease through Mendelian Randomization
Source: J Cardiovasc Dev Dis. 2024 Aug 27;11(9):264. doi: 10.3390/jcdd11090264 (PMC11432350; doi:10.3390/jcdd11090264)
Supplement: Supplementary file 1 [file jcdd-11-00264-s001.zip › jcdd-3128321-supplementary.pdf]

Supplemental Material

Supplemental Figure S1: Leave-one-out plots for CAD inference testing stratified for sex
Leave-one-out plots of male-specific MR of CAD with self-reported chest pain (A), back pain (B), facial pain (C), neck and shoulder pain (D), clinical chest pain (E) and female-specific MR of CAD with self-reported chest pain (F), back pain (G), facial pain (H), neck and shoulder pain (I), clinical chest pain (J).

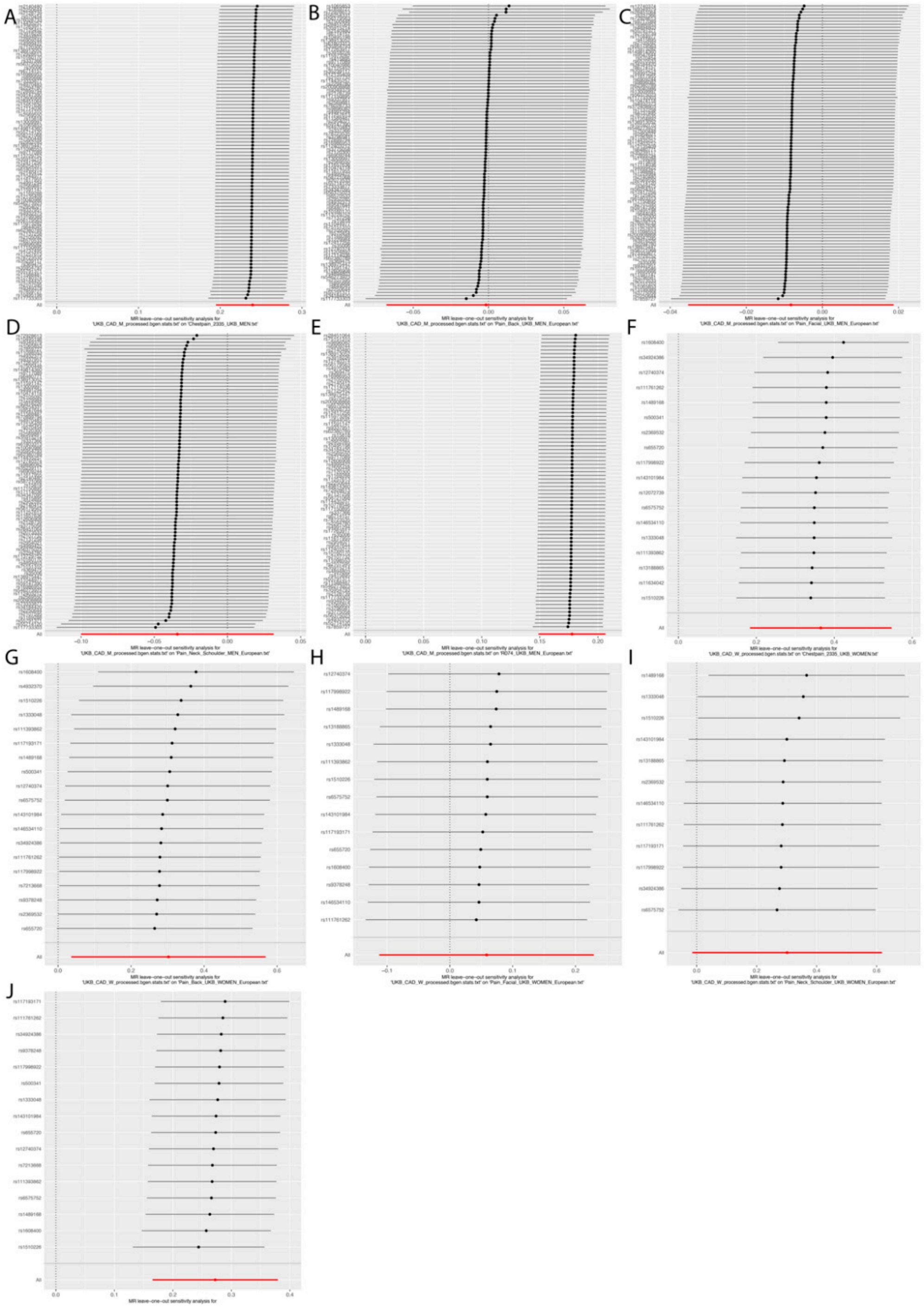

Supplemental Table S1: Sample sizes of the different traits

Sample sizes of participants with given trait in the UKBB study and the number controls used for linear mixed association testing. The last column describes the number of genome-wide significant (*p-value* < $5x10^{-8}$) genetic variants.

Trait	Cases	Controls	Genome-wide significant variants
Self-reported Chest Pain	72,296	382,284	771
Men	36,978	170,769	37
Women	35,318	211,515	56
Back Pain	117,394	300,804	5,871
Men	55,830	142,666	603
Women	61,564	158,138	1,494
Facial Pain	8,512	183,411	3,272
Men	2,545	86,837	23
Women	5,967	96,574	95
Neck and Shoulder Pain	105,534	183,411	5,466
Men	45,808	86,837	752
Women	59,726	96,574	963
Clinical Chest Pain	28,936	219,206	158
Men	14,608	195,289	34
Women	14,328	234,917	18
Atherogenic Cardiovascular Disease			
Coronary Artery Disease Men	22,323	181,500	2,349
Coronary Artery Disease Women	10,124	229,507	109

Supplemental Table S2: Genome-wide significant loci of different pain localizations stratified for sex

Top variants of sex-stratified GWAS of different pain localizations. Top clumped variant per genome-wide significant locus (p-value < $5x10^{-8}$) and its summary statistics with identified locus name. Loci names were defined by the nearest gene (< 50kb) to the top variant of given locus or mapped genes according to NHGRI-EBI GWAS Catalog (Bold). Variant position according to GRCh37 and Ensemble Gencode release 40 gene annotation. Chr: chromosome; BP; Base position; SE; standard error

Trait	Sex	Genetic Variant	Chr	BP	Effect Allele	Other Allele	Beta	SE	P-value	Locus
Self-reported Chest Pain	Men	rs113145710	1	37604815	G	GGA	-0.00793	0.00142	2.5×10^{-8}	ENSG00000284650
	Women	-	-	-	-	-	-	-	-	-
Clinical Chest Pain (ICD code: R07.4)	Men	rs55730499	6	161005610	C	T	-0.00837	0.00146	9.2×10^{-9}	**LPA**
	Women	rs66508877	13	19441912	G	A	-0.00675	0.00124	4.9×10^{-8}	TPTE2
Neck & Shoulder Pain	Men	rs13423867	2	53047108	G	A	0.0107987	0.00190964	1.6×10^{-8}	ENSG00000228033
		rs13086611	3	49385417	A	T	0.0114053	0.00201076	1.4×10^{-8}	**GPX1**
		rs13191118	6	17016502	C	G	0.0122455	0.00219896	2.6×10^{-8}	ENSG00000286631
		rs2424245	20	19649716	C	T	0.0165079	0.00279234	3.4×10^{-9}	**SLC24A3**
	Women	rs17491275	1	39672545	T	G	-0.0145776	0.00239839	1.2×10^{-9}	**MACF1**
		rs7555006	1	51480258	A	G	-0.00973802	0.00176652	3.5×10^{-8}	**MIR4421, CDKN2C**
		rs13135092	4	103198082	A	G	-0.0206285	0.00317972	8.7×10^{-11}	**SLC39A8**
		rs13135092	4	103198082	A	G	-0.0206285	0.00317972	8.7×10^{-11}	**SLC39A8**
		rs7105462	11	112912048	G	A	0.00997936	0.00177732	2×10^{-8}	**NCAM1**
		rs1079232	13	53906017	G	A	-0.0104526	0.00188917	3.1×10^{-8}	LINC00558
		rs12930744	16	71986545	G	A	0.0110144	0.00197217	2.3×10^{-8}	PKD1L3
		rs12453010	17	50316131	C	T	-0.0110824	0.00179311	6.4×10^{-10}	**CA10,LINC01982**
		rs7248205	19	10770305	C	T	0.0100383	0.0017833	1.8×10^{-8}	**ILF3**
Facial Pain	Men	-	-	-	-	-	-	-	-	-
	Women	rs117570769	9	75210309	C	T	-0.0230	0.00417	3.3×10^{-8}	RNU6-1228P
		rs12103300	16	8207573	T	C	0.00574	0.00104	3.1×10^{-8}	ENSG00000260003
Back Pain	Men	rs11708955	3	49540114	T	C	0.011089	0.0019902	2.5×10^{-8}	**DAG1**
	Women	rs7555006	1	51480258	A	G	-0.011063	0.00176627	3.8×10^{-10}	-
		rs1812249	1	112284581	G	A	-0.011674	0.00212193	3.8×10^{-8}	LINC02884
		rs7364668	1	150513534	A	G	0.0104293	0.0018223	1×10^{-8}	ECM1
		rs12464471	2	63686428	A	G	0.0114911	0.00210266	4.6×10^{-8}	-
		rs13107325	4	103188709	C	T	-0.0182354	0.00334148	4.8×10^{-8}	**SLC39A8**
		rs7654538	4	151201635	A	G	0.0102103	0.00176415	7.2×10^{-9}	
		rs1039325	5	30761421	T	G	-0.00998323	0.00177368	1.8×10^{-8}	ENSG00000287940
		rs2910576	5	57532748	A	T	0.0137766	0.00222658	6.1×10^{-10}	**PGAM1P1,PLK2**
		rs62381131	5	120211759	A	T	0.0101354	0.00177605	1.2×10^{-8}	ENSG00000251293
		rs2744937	6	34557246	C	T	-0.0157645	0.00250926	3.3×10^{-10}	SPDEF
		rs10282983	8	69590554	C	T	-0.0133516	0.00207072	1.1×10^{-10}	**C8orf34**
		rs79164994	8	130719443	A	G	0.0135706	0.00211749	1.5×10^{-10}	ADCY8
		rs7942857	11	112881906	T	C	-0.0101282	0.00178917	1.5×10^{-8}	ENSG00000288070
		rs2955527	12	23973837	T	G	-0.0142014	0.00204568	3.9×10^{-12}	SOX5
		rs12453010	17	50316131	C	T	-0.0103483	0.00179226	7.8×10^{-9}	**CA10,LINC01982**
		rs570217967	18	60247998	C	G	0.012492	0.00224208	2.5×10^{-8}	-

Supplemental Table S3: Instrumental variables of CAD for men and women

IVs included for MR analysis. IVs were included with CAD GWAS p-value < $5x10^{-6}$ after clumping at $R^2 < 0.001$ with F-statistic > 10. EAF: effect allele frequency, SE: standard error.

Sex	SNP	Effect Allele	Other Allele	EAF	Beta	SE	P-value	F-statistic
Men	rs4970483	C	G	0.7334	0.0052	0.0011	3.1E-06	20.4
	rs11591147	G	T	0.9825	0.0285	0.0037	3.2E-14	33.5
	rs17114036	A	G	0.9080	0.0112	0.0017	5.4E-11	28.9
	rs12740374	G	T	0.7785	0.0119	0.0012	1.5E-23	44.4
	rs11588487	G	T	0.6904	-0.0053	0.0011	8.5E-07	21.6
	rs12135409	C	T	0.7844	-0.0057	0.0012	2.5E-06	20.6
	rs11204892	A	G	0.1598	-0.0063	0.0014	2.8E-06	20.5
	rs61803313	C	A	0.6473	0.0052	0.0011	2.6E-06	20.6
	rs10495198	T	C	0.2852	-0.0082	0.0011	5.8E-14	33.1
	rs16986953	G	A	0.9320	-0.0105	0.0020	8.9E-08	23.5
	rs2140412	G	C	0.3237	0.0050	0.0011	2.6E-06	20.6
	rs6714157	A	G	0.5301	-0.0054	0.0010	5.8E-08	23.8
	rs13009997	C	T	0.8290	0.0065	0.0013	7.3E-07	21.7
	rs72928613	T	A	0.8713	-0.0117	0.0015	1.7E-15	35.2
	rs372408903	A	C	0.8703	0.0070	0.0015	1.6E-06	21.0
	rs4894803	A	G	0.5888	-0.0047	0.0010	3.8E-06	20.3
	rs115973282	C	T	0.9524	0.0106	0.0023	4.6E-06	20.1
	rs138913052	C	T	0.9744	-0.0151	0.0032	2.2E-06	20.8
	rs35954793	C	A	0.7781	-0.0063	0.0012	1.7E-07	23.0
	rs3775058	A	T	0.2132	0.0065	0.0012	6.4E-08	23.7
	rs1229984	T	C	0.0253	-0.0147	0.0032	3.8E-06	20.3
	rs11098553	A	G	0.3238	0.0057	0.0011	9.5E-08	23.4
	rs114435257	T	G	0.9427	-0.0100	0.0021	2.7E-06	20.6
	rs58721068	A	G	0.8562	-0.0101	0.0014	8E-13	31.6
	rs3796581	A	G	0.8155	0.0067	0.0013	1.2E-07	23.3
	rs75151310	A	G	0.9502	-0.0119	0.0023	1.4E-07	23.1
	rs17263917	G	A	0.8468	0.0070	0.0014	2.5E-07	22.6
	rs10040986	T	A	0.9165	-0.0084	0.0018	3.2E-06	20.4
	rs35006	C	G	0.6903	0.0052	0.0011	9.2E-07	21.5
	rs9717089	T	C	0.6154	0.0052	0.0010	5.5E-07	22.0
	rs10474118	T	C	0.6592	0.0049	0.0010	3.1E-06	20.4
	rs62380789	A	C	0.9145	-0.0083	0.0018	3.1E-06	20.4
	rs337366	G	A	0.5031	-0.0057	0.0010	1.2E-08	25.0
	rs2569881	G	A	0.8714	0.0068	0.0015	4.4E-06	20.1
	rs9349379	A	G	0.5942	-0.0094	0.0010	8.2E-21	41.4
	rs6909244	T	C	0.6716	-0.0049	0.0010	2.6E-06	20.6
	rs644045	A	G	0.3883	-0.0059	0.0010	7E-09	25.4
	rs72899794	G	A	0.9019	0.0078	0.0017	2.6E-06	20.6

	rs13199389	G	A	0.5426	0.0046	0.0010	3.6E-06	20.3
	rs369475	T	C	0.0656	0.0096	0.0020	1.5E-06	21.1
	rs117150895	G	A	0.9779	0.0180	0.0034	8.3E-08	23.5
	rs2105092	G	A	0.7087	0.0076	0.0011	2.3E-12	30.9
	rs6570533	G	A	0.4418	0.0045	0.0010	4.8E-06	20.0
	rs2294780	C	G	0.7056	-0.0050	0.0011	4.9E-06	20.0
	rs117733303	A	G	0.9816	-0.0446	0.0037	3.3E-34	54.3
	rs2073533	T	C	0.3202	-0.0051	0.0011	1.8E-06	20.9
	rs2107595	G	A	0.8472	-0.0087	0.0014	2.9E-10	27.7
	rs2107732	G	A	0.9072	0.0078	0.0017	4.8E-06	20.0
	rs34347095	G	T	0.8692	0.0067	0.0015	4.6E-06	20.1
	rs56179563	G	A	0.6114	0.0055	0.0010	5.6E-08	23.8
	rs3918226	C	T	0.9188	-0.0097	0.0018	1.3E-07	23.2
	rs59147390	T	C	0.8899	0.0079	0.0016	6E-07	21.9
	rs4737495	G	T	0.4019	0.0046	0.0010	4.9E-06	20.0
	rs73333877	G	T	0.7731	-0.0056	0.0012	1.7E-06	21.0
	rs76028733	A	T	0.9633	-0.0126	0.0027	2.3E-06	20.7
	rs4999422	A	C	0.3013	0.0055	0.0011	4.2E-07	22.2
	rs2954021	A	G	0.4959	0.0049	0.0010	5.7E-07	21.9
	rs138975447	C	T	0.9852	-0.0221	0.0044	4.2E-07	22.2
	rs7859727	C	T	0.5108	-0.0163	0.0010	2.4E-61	74.4
	rs2140480	T	C	0.2858	0.0070	0.0011	1.7E-10	28.1
	rs11257613	G	A	0.4861	0.0047	0.0010	2E-06	20.8
	rs9337951	G	A	0.6551	-0.0060	0.0011	1.4E-08	24.9
	rs10160170	A	G	0.8882	0.0093	0.0016	3.4E-09	26.0
	rs6480771	T	C	0.5876	-0.0047	0.0010	2.2E-06	20.8
	rs2250644	C	T	0.6590	-0.0055	0.0010	1.4E-07	23.1
	rs4991294	T	G	0.5691	-0.0055	0.0010	4.7E-08	24.0
	rs7120300	C	T	0.2629	0.0052	0.0011	3.7E-06	20.3
	rs415895	C	G	0.3536	-0.0052	0.0010	3.5E-07	22.4
	rs570454	T	G	0.2981	0.0053	0.0011	1E-06	21.4
	rs7125452	G	A	0.5130	-0.0045	0.0010	4.8E-06	20.0
	rs2128739	A	C	0.2797	0.0077	0.0011	3.1E-12	30.7
	rs15818	A	G	0.5914	-0.0050	0.0010	7.2E-07	21.7
	rs12417256	A	C	0.6044	-0.0047	0.0010	2.9E-06	20.5
	rs11044977	C	T	0.6148	-0.0061	0.0010	2.1E-09	26.3
	rs7137258	C	A	0.9450	-0.0124	0.0022	1.6E-08	24.8
	rs35239117	A	T	0.9295	0.0092	0.0019	1.8E-06	20.9
	rs10778546	A	T	0.6049	0.0046	0.0010	4.7E-06	20.1
	rs4766578	T	A	0.4959	0.0062	0.0010	3.1E-10	27.7
	rs1169288	A	C	0.6857	-0.0066	0.0011	4.9E-10	27.4
	rs112403212	C	T	0.8600	-0.0068	0.0014	2.1E-06	20.8
	rs9547644	A	C	0.8680	-0.0068	0.0015	4.1E-06	20.2
	rs11617955	T	A	0.8872	0.0090	0.0016	1.3E-08	25.0
	rs149814360	T	C	0.9787	-0.0172	0.0036	2.1E-06	20.8

	rs546273923	T	C	0.8399	-0.0070	0.0015	2.6E-06	20.6
	rs56375023	G	A	0.7632	0.0065	0.0012	2.1E-08	24.6
	rs34184420	G	T	0.5466	0.0067	0.0010	1.1E-11	29.9
	rs4932373	A	C	0.6739	-0.0075	0.0011	9.3E-13	31.5
	rs7500448	A	G	0.7461	0.0061	0.0011	7.6E-08	23.6
	rs11657636	C	T	0.3022	-0.0056	0.0011	2.3E-07	22.7
	rs200908868	A	G	0.5082	-0.0052	0.0010	1.5E-07	23.0
	rs55714120	G	T	0.6646	-0.0076	0.0010	5.3E-13	31.8
	rs2952285	G	A	0.3797	-0.0048	0.0010	2.8E-06	20.5
	rs9896082	A	C	0.9366	-0.0100	0.0021	1.5E-06	21.1
	rs12606908	C	T	0.6954	0.0050	0.0011	3.7E-06	20.3
	rs895666	A	G	0.4015	-0.0051	0.0010	4E-07	22.2
	rs78723535	C	T	0.9853	-0.0188	0.0041	4.6E-06	20.1
	rs55791371	A	C	0.8818	0.0106	0.0015	3.6E-12	30.6
	rs2569550	T	C	0.4068	-0.0055	0.0010	3.6E-08	24.2
	rs35915214	A	C	0.3743	-0.0047	0.0010	4.2E-06	20.2
	rs56254331	A	C	0.8311	-0.0078	0.0013	3.8E-09	25.9
	rs1065853	G	T	0.9194	0.0157	0.0018	4.8E-18	38.3
	rs113729752	T	C	0.8512	-0.0069	0.0014	1.1E-06	21.4
	rs28451064	G	A	0.8677	-0.0121	0.0015	4.1E-16	35.9
Women	rs12072739	A	G	0.7758	-0.0032	0.0007	4.8E-06	20.9
	rs12740374	G	T	0.7789	0.0035	0.0007	5.5E-07	25.1
	rs2369532	A	T	0.2416	0.0032	0.0007	3.4E-06	21.6
	rs500341	T	C	0.3315	0.0029	0.0006	3.7E-06	21.4
	rs111393862	A	T	0.9408	-0.0058	0.0013	3.9E-06	21.3
	rs34924386	C	T	0.9521	0.0064	0.0014	2.6E-06	22.1
	rs655720	C	T	0.2801	-0.0032	0.0006	1.2E-06	23.6
	rs686235	G	C	0.4290	0.0028	0.0006	2.5E-06	22.2
	rs13188865	G	T	0.8360	0.0039	0.0008	7.3E-07	24.5
	rs9378248	G	A	0.6611	-0.0029	0.0006	2.7E-06	22.0
	rs146534110	G	T	0.9864	-0.0133	0.0025	1.3E-07	27.9
	rs1510226	T	C	0.9806	-0.0135	0.0021	2.3E-10	40.2
	rs111761262	T	C	0.8641	0.0043	0.0009	5.9E-07	24.9
	rs117998922	G	A	0.9797	-0.0103	0.0021	1.6E-06	23.0
	rs1333048	A	C	0.5003	-0.0047	0.0006	5.1E-16	65.8
	rs143101984	G	A	0.9836	-0.0123	0.0026	1.8E-06	22.8
	rs1608400	T	C	0.1476	0.0042	0.0008	5.4E-07	25.1
	rs117193171	T	C	0.9633	-0.0071	0.0016	4.2E-06	21.2
	rs6575752	T	C	0.8453	-0.0041	0.0008	4.9E-07	25.3
	rs11634042	C	T	0.5626	0.0031	0.0006	1.7E-07	27.3
	rs4932370	G	A	0.6643	-0.0029	0.0006	3.1E-06	21.7

	rs7213668	G	A	0.7922	-0.0034	0.0007	2.1E-06	22.5
	rs1489168	T	A	0.6672	0.0030	0.0006	2.6E-06	22.1

Supplemental Table S4: Results of power analysis
Power expressed as minimum Odds Ratio to obtain for 80% power (alpha: 0.05) are denoted. Proportion of Cases according to sample sizes described in Suppl. Table 1. Variance calculated using the sum of variances explained of individual IVs of CAD.

Sex	Trait	Proportion of cases (outcome)	R^2_{xz}	Odds Ratio required
Men	Chest Pain	0.178	0.0090	1.18
	Clinical Chest Pain	0.070	0.0090	1.26
	Back Pain	0.281	0.0090	1.15
	Facial Pain	0.028	0.0090	1.60
	Neck and Shoulder Pain	0.345	0.0090	1.18
Women	Chest Pain	0.143	0.0025	1.32
	Clinical Chest Pain	0.057	0.0025	1.49
	Back Pain	0.280	0.0025	1.29
	Facial Pain	0.058	0.0025	1.77
	Neck and Shoulder Pain	0.382	0.0025	1.32

Supplemental Table S5: Example of pleiotropy induced by genetic variant in LPA locus
Genetic association of the LPA locus is shared in both CAD and chest pain, suggesting potential horizontal pleiotropy. MR Egger intercept analysis with CAD as exposure and self-reported chest pain as outcome reveals pleiotropy. Exclusion of the IV in this locus resolves this.

Condition	Egger Intercept	SE	P-value
Including LPA locus	-0.00079	0.00037	0.03685
Excluding LPA locus	-0.00067	0.00069	0.34382

Supplemental Table S6: MR analyses and sensitivity analyses results of sex-stratified causal inference

MR IVW results and MR Weighted Median results expressed in Odds Ratio and 95% confidence interval, MR Egger intercept results, and heterogeneity results for the different traits being compared for men and women separately.

Sex	Trait	Inverse variance weighted		Weighted Media		Sensitivity analyses				P-value comparison to women
		Beta (SE)	*P-value*	*Beta (SE)*	*P-value*	*MR-Egger non-zero intercept p-value*	*Cochran Q-value p-value*	*MR-PRESSO Global test p-value*	*Steiger directionality test p-value*	
Men	*Self-reported Chest Pain*	0.240 (0.023)	$1.16x10^{-25}$	0.279 (0.035)	$2.79x10^{-15}$	0.052	0.909	0.948	$9.40x10^{-208}$	0.189
	Clinical Chest Pain	0.178 (0.015)	$1.58x10^{-33}$	0.189 (0.023)	$6.17x10^{-16}$	0.986	0.996	0.999	$2.67x10^{-189}$	0.094
	Back Pain	-0.002 (0.034)	0.961	0.002 (0.052)	0.971	0.469	0.254	0.084	$6.97x10^{-244}$	0.029
	Neck and Shoulder Pain	-0.034 (0.034)	0.308	-0.065 (0.055)	0.231	0.937	0.596	0.441	$1.47x10^{-174}$	0.041
	Facial Pain	-0.008 (0.014)	0.548	-0.009 (0.021)	0.680	0.579	0.998	0.997	$5.20x10^{-267}$	0.458
Women	*Self-reported Chest Pain*	0.365 (0.093)	$8.13x10^{-5}$	0.467 (0.122)	$1.21x10^{-4}$	0.290	0.226	0.271	$2.34x10^{-29}$	
	Clinical Chest Pain	0.272 (0.054)	$6.26x10^{-7}$	0.251 (0.078)	$1.35x10^{-3}$	0.812	0.879	0.919	$1.37x10^{-27}$	
	Back Pain	0.302 (0.136)	0.026	0.308 (0.193)	0.111	0.978	0.415	0.469	$7.23x10^{-34}$	
	Neck and Shoulder Pain	0.301 (0.161)	0.062	0.424 (0.211)	0.044	0.648	0.950	0.964	$6.91x10^{-22}$	
	Facial Pain	0.058 (0.087)	0.503	0.043 (0.111)	0.697	0.942	0.998	0.997	$2.60x10^{-21}$	
